# Optimizing hospice care: A scoping review of staffing strategies to enhance practice

**DOI:** 10.1016/j.ijnsa.2026.100553

**Published:** 2026-05-01

**Authors:** Victoria Kao, Vidhi Patel, Kiana Aghakhani Barfeh, Philip Pranajaya, Jayne Kang, Madeleine Wong, Hannah McDonald, Amanda Ross-White, Craig E. Goldie, Danielle Kain, Sarah Moore-Vasram, Catherine L. Goldie

**Affiliations:** a School of Nursing, Queen’s University, Kingston, ON, Canada; b Providence Care Hospital, Kingston, ON, Canada; c Faculty of Health Sciences, Queen’s University, Kingston, ON, Canada; d Bracken Health Sciences Library, Queen’s University, Kingston, ON, Canada; e Division of Palliative Medicine, Department of Medicine, Queen’s University, Kingston, ON, Canada

**Keywords:** Hospice care, Health workforce, Personnel staffing and scheduling, Quality improvement, Volunteers

## Abstract

**Background:**

Strategic hospice staffing is essential to optimize patient care and strengthen workforce performance and retention, particularly as aging populations and rising rates of life-limiting illnesses increase the demand for end-of-life care. Despite growing recognition of staffing challenges in hospice care, there is limited synthesis of evidence on effective models and practices that support high-quality, sustainable care delivery.

**Objective:**

This scoping review aimed to synthesize evidence on workforce characteristics and strategies that support sustainable, high-quality hospice care.

**Information sources:**

Literature published after 2013 was searched in Ovid MEDLINE, EBSCOhost CINAHL, Ovid Embase, and Google Scholar to capture both peer-reviewed and grey literature sources relevant to hospice staffing and care quality.

**Methods:**

The review followed the PRISMA-ScR framework and was guided by the Arksey and O’Malley five-stage methodology. Search terms reflected three dimensions: 1) adult hospice care, 2) hospice staffing, and 3) the quality and effectiveness of care. Articles were included if they discussed hospice or palliative care staffing models, workforce characteristics, or operational performance metrics.

**Results:**

Forty-seven articles were included (33 peer-reviewed publications and 14 grey literature sources), representing hospice and palliative care settings across 11 countries, with the United States most frequently studied. Core strategies to enhance workforce sustainability and patient care included staffing composition and competency, key care approaches, and volunteer integration Findings highlighted that having an appropriately structured and well-prepared workforce is critical for ensuring quality care, while strategies that promote staff well-being and streamline processes further enhance service delivery. Collectively, these themes point to guiding principles for hospice staffing: adaptable, patient-responsive models; strong interdisciplinary collaboration; ongoing training and support; and the strategic use of volunteers and technology.

**Conclusions:**

This review synthesizes international evidence on hospice staffing and identifies common principles that can guide practice. Taken together, these practical insights highlight an urgent need for rigorous evaluation to move beyond fragmented approaches and establish sustainable, equitable, and patient-centred staffing standards. Developing and implementing such standards is essential not only for workforce stability, but also for ensuring high-quality, compassionate care for patients and families at the end of life.

**Registration:**

Not registered.

**Social media abstract:**

Interdisciplinary, flexible staffing models improve hospice care quality and support workforce sustainability

What is already known

• Hospice care improves quality of life and patient satisfaction; demand is rising.
• No standardized, peer-reviewed strategies exist for effective hospice staffing.

What this paper adds

• Flexible, acuity-based staffing improved patient-centred hospice care.
• Training, volunteers, and technology boost workforce capacity and outcomes.
• Evidence-based principles to support adaptable and equitable hospice staffing.

Alt-text: Unlabelled box dummy alt text

## 1 Background

Hospice care provides comprehensive end-of-life support, emphasizing comfort, dignity, and quality of life rather than curative treatment (National Insitute on Aging, 2021). Compared to hospital-based end-of-life care, hospice services enhance patient and family satisfaction while reducing intensive medical interventions (Kelley et al., 2013). With aging populations, rising rates of life-limiting illness, and increasing awareness of hospice services, demand for care continues to grow (Bone et al., 2018; Etkind et al., 2017; Lupu et al., 2018; World Health Organization, 2020). Meeting this demand requires not only physical infrastructure but a well-supported workforce sustained by aligned resources, professional expertise, ongoing education, and enabling policy frameworks (Butler et al., 2024).

### 1.1 Staffing challenges

Despite its crucial importance, hospice staffing faces persistent challenges. High turnover is common, particularly among nurses, who constitute a substantial portion of the workforce. Hospice care staff frequently report intent or thoughts of leaving the profession (Head et al., 2019; Stone et al., 2013), with annual turnover among nursing and administrative hospice staff in the United States averaging 27.9 % as of 2023 (Hospital and Healthcare Compensation Service, 2024). Shorter retention is linked to heavy workloads, emotional burnout, limited support and recognition, inadequate compensation and benefits, and challenges with patients and their families (Head et al., 2019; Luo et al., 2013; Stone et al., 2013). Recruitment and retention are further complicated by geographical location, with rural settings presenting additional barriers to workforce stability and continuity of care (Accreditation Canada, 2023; Northern Ontario School of Medicine, 2019; Wan et al., 2023).

Another barrier is the limited specialized training in palliative and end-of-life care. Many healthcare professionals, particularly nurses, have insufficient formal education on pain and symptom management, psychological support, and communication strategies for discussing prognosis and death (American Nurses Association Professional Issues Panel, 2017; Dudley et al., 2018; Pawlow et al., 2018). Providers are also under pressure to continuously adapt their care approaches to meet the growing complexity of patient needs (Greenstein et al., 2019; Gurian et al., 2019; Newton and McVicar, 2014;; Tupala et al., 2020). Inadequate preparation also contributes to low staff remuneration and volunteer retention, which in turn can lead to poorer patient outcomes and gaps in culturally competent care (Claxton-Oldfield and Jones, 2013; Pesut et al., 2014; Szeliga and Mirecka, 2018). This lack of preparedness increases stress and resource allocation towards on-the-job training, hindering effective service delivery and patient access (Dudley et al., 2018; Pesut et al., 2014).

### 1.2 Research gap and study rationale

Evidence across healthcare settings consistently demonstrates that appropriate staffing and strong interdisciplinary collaboration are critical for continuity, symptom management, and patient satisfaction (Aiken et al., 2021; Ball and Griffiths, 2022; Registered Nurses' Association of Ontario, 2017; Yoon, 2022). However, ensuring an adequately staffed and well-supported hospice workforce remains a persistent challenge; one exacerbated by the growing demand for services and the lasting impact of the COVID-19 pandemic (Lupu et al., 2018; Schneider et al. 2022; Wakefield et al., 2021).

In this context, identifying and evaluating effective staffing strategies is essential to meet the evolving needs of hospice care. A brief search in the Ovid MEDLINE and EBSCOhost CINAHL databases (March 10, 2025) identified seven reviews: five examined factors influencing hospice performance (He et al., 2020; Moore et al., 2016; Newbould et al., 2022; Pesut et al., 2014; Thavaraj and Gillett, 2019), and two explored workforce challenges (Adia, 2023; Papworth et al., 2024). While these reviews provide valuable insights into specific aspects of workforce performance, none synthesize strategies to strengthen hospice staffing models, improve service efficiency, and ultimately enhance patient and family care.

The current study used a scoping review methodology to synthesize evidence on workforce characteristics and strategies that optimize the sustainability and quality of hospice care. As the demand for end-of-life care continues to grow, the findings of this review aim to inform broader discussions on healthcare workforce planning, and evidence-based guidelines.

## 2 Methods

This scoping review followed the 5-stage methodological framework described by Arksey and O’Malley (2005) and is reported in accordance with the Preferred Reporting Items for Systematic Reviews and Meta-Analysis Extension for Scoping Reviews (PRISMA-ScR) Checklist (Arksey and O'Malley, 2005; Tricco et al., 2018).

### 2.1 Stage 1: identifying the research question

The following research question was used to guide the literature search: What does the existing literature reveal about workforce characteristics and strategies that support sustainable, high-quality hospice care?

### 2.2 Stage 2: identifying relevant studies

A comprehensive search strategy was developed based on the three-step approach outlined in the JBI Manual for Evidence Synthesis (Peters et al., 2020). Our search was conducted across four databases, including Ovid MEDLINE, EBSCOhost CINAHL, Ovid Embase, and Google Scholar.

An initial search was conducted in Ovid MEDLINE and EBSCOhost CINAHL to identify relevant studies and refine the search strategy. We collaborated with the Health Sciences Research Librarian at Queen’s University (AR-W) to develop and categorize search terms based on three dimensions: 1) the context and population of interest (adult hospice care), 2) hospice staffing, and 3) the outcome of interest (quality and outcomes of care). Search terms within each dimension were combined using the Boolean *OR* operator, and each dimension was separated by the Boolean *AND* operator, as outlined in Table 1. The results from this search were imported into Covidence for the screening of titles, abstracts, and index terms. This led to a revised search strategy, which was applied to Ovid MEDLINE, EBSCOhost CINAHL, and Ovid Embase. The search was conducted on April 25, 2025. To ensure comprehensive coverage, reference lists of included reviews were manually screened for additional sources, and a targeted Google Scholar search was conducted to capture grey literature relevant to the research question.

**Table 1:** Key search terms.

	Dimension 1: Context and Population of Interest	Dimension 2: Hospice Staffing	Dimension 3: Quality and Effectiveness of Care
Initial search terms	“hospices” OR “hospice care” OR “hospice and palliative care nursing”	“models of care” OR “staffing”	"quality of health care”
Revised search terms	“hospices” OR “hospice care” OR “hospice and palliative care nursing” OR “hospice nurses” OR “hospice nursing”	“personnel staffing and scheduling” OR “personnel management” OR “health care personnel management skill mix” OR “models of care”	“outcome assessment” OR “health care” OR “quality of health care”

### 2.3 Stage 3: study selection

**Fig. 1:**
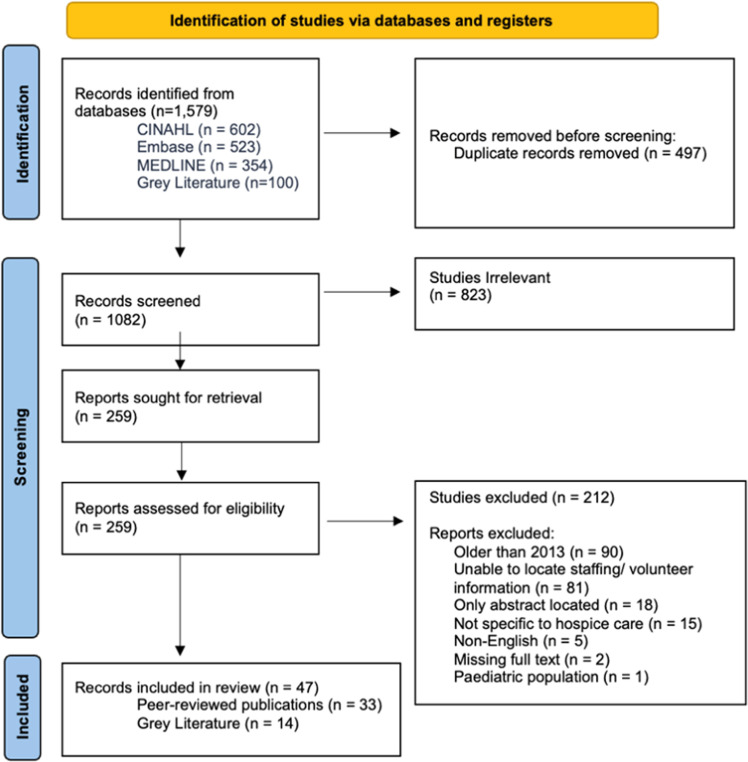
PRISMA-ScR flow chart for study selection.

Out of the 1579 articles identified from the search, 497 duplicates were automatically removed by Covidence. The remaining 1082 articles were screened using a two-step selection process guided by the eligibility criteria outlined in Table 2. A PRISMA-ScR summary of the study selection process can be found in Fig. 1.

**Table 2:** Eligibility criteria.

Criterion	Inclusion	Exclusion
Types of evidence sources	• Peer-reviewed research, including surveys, qualitative studies, reviews, retrospective reviews, cohort studies, and mixed methods studies • Non-empirical sources such as narrative reviews • Completed clinical trials or workforce interventions providing data on staffing workforce outcomes, or care effectiveness • Empirical grey literature, such as accreditation reports, policy documents, and guidelines that address hospice staffing	• Non-empirical sources such as editorials, opinion pieces, and commentaries • Conference abstracts without full articles • Studies and organizational reports unrelated to hospice or palliative workforce issues
Populations	• Adults (18+) receiving hospice care • Hospice staff (nurses, physicians, aides, social workers, volunteers, allied health), and families when linked to staffing issues	• Paediatric or neonatal intensive care unit patients • Non-human populations
Concept	• Studies investigating hospice staffing and workforce issues, including: ◦ Staffing models, ratios, and skill mix ◦ Roles, responsibilities, and interdisciplinary collaboration ◦ Recruitment, retention, training, workload, and burnout ◦ Volunteer roles and integration ◦ Operational or organizational metrics tied to staffing and quality of care	• Studies focused only on prognostic/admission tools or patient eligibility • Studies that mentioned staffing superficially without analysis • Interventions unrelated to hospice workforce or service delivery
Context	• Global studies, any country • Publications from 2013 to March 2025 • Healthcare settings providing hospice services: ◦ Inpatient hospices ◦ Hospital-based hospice or palliative care units ◦ Home hospice programs ◦ Community-based hospice programs	• Non-healthcare or non-palliative contexts • Publications before 2013 or after March 2025

Seven reviewers (KA, JK, HM, PP, VP, DT, MW) independently screened all titles and abstracts in Covidence, classifying each article as “Yes,” “No,” or “Maybe.” Discrepancies were discussed among reviewers to reach consensus on which articles should proceed to full-text review. A total of 823 articles were excluded, primarily due to a lack of hospice-specific information, a focus on paediatric or Neonatal Intensive Care Unit patients, being published before 2013, and being in a format deemed inadmissible such as abstracts and commentaries.

The same reviewers (KA, JK, HM, PP, VP, DT, MW) then conducted full-text screening. The remaining 259 articles were divided among the group, with each article independently assessed by two reviewers. Any disagreements were resolved through group discussions with the senior investigator (CLG). Following this rigorous process, 47 articles that met the inclusion criteria were selected for data extraction and analysis.

### 2.4 Stage 4: charting the data

An evidence extraction template was developed in Covidence to guide eight reviewers (KA, CG, JK, VK, HM, PP, VP, DT) in capturing relevant details to support comparison across studies and understand how each study contributed to the synthesis. The template documented publication details (authors, year, and country), article type (methodological approach or grey literature), population characteristics (participant groups, sample sizes, and relevant contextual factors), and procedural information (aims, methods, and analytic strategies). It also captured key findings related to workforce composition, training approaches, staffing challenges, and volunteer involvement, as well as recommendations for practice and policy. These charting components and themes were deliberately selected to reflect core elements of scoping reviews and allowed the review team to map how hospice staffing is conceptualized, operationalized, and evaluated across diverse settings.

Sex and gender were not used as variables in this scoping review, as the included studies primarily focused on organizational and workforce-level factors rather than individual participant characteristics. Where studies reported sex or gender composition (e.g., of staff or patients), this information was extracted but was not as focus of analysis. We acknowledge the limited reporting of sex and gender considerations in the included literature.

### 2.5 Stage 5: collating, summarizing and reporting the results

After data extraction was completed, the thematic analysis was conducted by a single reviewer (VK), who systematically reviewed all extracted data to identify recurrent patterns related to hospice staffing. The reviewer began by generating initial descriptive codes directly from the extracted material, iteratively refining these codes as similarities, differences, and conceptual links became apparent across studies. These codes were then grouped into higher-level categories, allowing the reviewer to move from descriptive coding to the development of more interpretive themes. The resulting themes of staffing composition and competency, key care approaches, and volunteer integration, were finalized after consultation with the senior investigator (CLG) and repeated comparison back to the extracted data to ensure they accurately summarized the breadth of findings and recommendations across sources. Once the thematic framework was established, each article was mapped to the final themes to enhance transparency, and this mapping was added to Appendix A to allow readers to verify how extracted data informed the synthesis.

## 3 Results

**Fig. 2:**
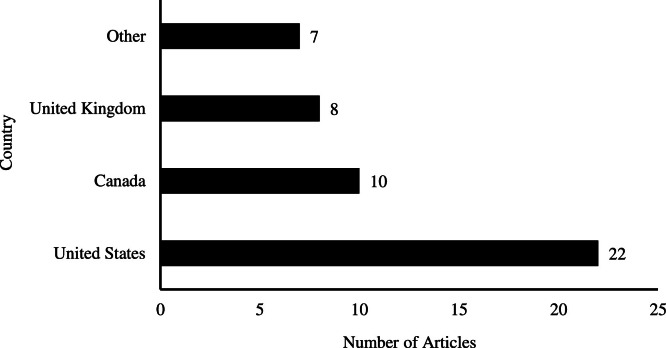
Number of articles by country of publication (*n* = 47). “Other” includes one article each from Germany, Ireland, Poland, South Korea, and Denmark/Australia. For five articles involving multiple countries, the country of publication is used. These include four reviews (Pesut et al., 2014; Blacker et al., 2016; Walshe et al., 2023; Terjung et al., 2024), and one piece of grey literature (World Health Organization, 2016). Further details on contributing countries can be found in Appendix A.

**Fig. 3:**
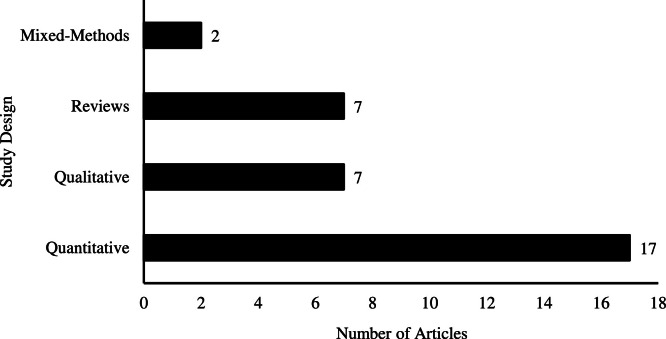
Number of peer-reviewed publications by research design (*n* = 33).

The search strategy identified 47 articles from 11 countries, with the majority coming from the United States (*n* = 22), followed by Canada (*n* = 10), United Kingdom (*n* = 8), one each from Germany, Ireland, Italy, Poland, South Korea, Switzerland, and one from both Denmark and Australia; as shown in Fig. 2. Of these articles, 33 are peer-reviewed publications and 14 are grey literature. Within the peer-reviewed literature, study designs were grouped into four distinct, non-overlapping categories, as shown in Fig. 3: quantitative studies (*n* = 17), qualitative studies (*n* = 7), reviews (*n* = 7), and mixed-methods studies (*n* = 2). Quantitative studies included survey, observational (including cohort and retrospective), and a quasi-experimental designs. All review types were combined into one category, including an individual case study that functioned as a non-empirical, descriptive paper. The grey literature sources provide guidelines and recommendations for hospice service delivery, program development, and staffing to different capacities.

Although this review focuses on hospice workforce characteristics and staffing, we included studies centred on patients, family caregivers, and volunteers when they offered insight into workforce issues or showed how staffing structures and practices shape the sustainability and quality of hospice care. The peer-reviewed publications therefore spanned five population groups: patients, hospice staff, volunteers, hospice management, and family caregivers. Among the included studies, 11 focused on patients, nine on staff, six on patients and family caregivers in conjunction with at least one other population category, four focused on volunteers, two on management, and one on hospice staff and family caregivers. Across the 25 primary investigations, sample sizes ranged from 13 participants in Kang et al. (2021) to 43,200 participants in Cornell et al. (2023).

The literature highlights three core strategies to enhance workforce sustainability and patient care, which are synthesized in the remainder of this section: 1) the composition and competency of staff, 2) key care approaches, and 3) the effective integration of volunteers.

1) *Staffing Composition and Competency*
  Adequate staffing ratios and a balanced mix of multidisciplinary skills were frequently identified as essential factors for ensuring quality hospice care delivery. While most articles provided high-level reasoning, one secondary analysis on the symptom control of 992 adult patients from residential hospices in Italy provided exact ratios for optimal staffing: 5.5–6.5 patients to 1 physician, 1.5–2.7 patients to 1 nurse, and 4.1–6.3 patients to 1 nurse assistant (Artico et al., 2022). Since these ratios may not generalize to home-based programs, rural settings, or systems with different regulatory structures, their applicability should be interpreted with caution. Beyond the recognition that physicians and nurses must collaborate effectively when delivering end-of-life care, multiple studies also highlighted the incorporation of a diverse range of specialists, including social workers, chaplains, psychiatrists, therapists, spiritual care providers, and family caregivers (Blacker et al., 2016; Braveman et al., 2015; Kang et al., 2021; McInnerney et al., 2021).
  In addition to optimizing hospice care team composition, several articles highlighted the need for improved training systems and ongoing professional development to enhance staff confidence, reduce burnout, and support high-quality care. Many found that high-performing hospice care programs were associated with the prioritization of educational initiatives, such as spiritual care training programs to reduce compassion fatigue and enhance holistic care, expanded palliative content on nursing licensure exams, Objective Structured Clinical Examinations to evaluate key competencies and skills, and targeted instruction on topics such as personal protective equipment, telehealth, and mental health (American Nurses Association Professional Issues Panel, 2017; Dudley et al., 2018; Kang et al., 2021; Szeliga and Mirecka, 2018). Studies consistently linked such training to improved retention and more positive patient and family care experiences (Dudley et al., 2018; Head et al., 2019; Kang et al., 2021; Pawlow et al., 2018).
2) *Key Care Approaches*
  Rather than representing distinct models of care, the literature described a set of key approaches that support effective hospice staffing, highlighting core characteristics that high-performing hospices incorporate to address workforce needs and enhance patient care.
  2.1) Patient-centred care emerged across multiple studies as a foundational approach shaping hospice staffing by emphasizing individualized care plans, shared decision-making, and alignment with patient and family priorities (Braveman et al., 2015; Cornell et al., 2023; Grant and Scott, 2015; World Health Organization, 2020). In hospice settings, this approach differs from patient-centred care in acute care settings by explicitly incorporating anticipatory planning, family-led decision-making, and holistic psychosocial support as patients decline, which requires strong interdisciplinary communication and coordination (Bhavsar et al., 2017; Braveman et al., 2015; Cornell et al., 2023). Families were central contributors to decisions, ongoing assessment of needs, and goal setting, particularly in situations where caregivers or volunteers supported daily care demands (Melekis et al., 2023; Walshe et al., 2021). By reducing unnecessary interventions and optimizing resource allocation, studies reported that patient-centred approaches improved continuity, streamlined workloads, and enhanced caregiver satisfaction and emotional preparedness (Accreditation Canada, 2019; Braveman et al., 2015; Cornell et al., 2023; Grant and Scott, 2015).
  2.2) Community-based approaches rely on trained volunteers, family caregivers, and local organizations to supplement formal staffing and curate a “home away from home” (Grant and Scott, 2015; Melekis et al., 2023; Walshe et al., 2021). Two approaches highlighted in the literature are the Social Hospice Model, which offers 24-hour, community-supported care for people who cannot be safely supported at home (Melekis et al., 2023), and the Cottage Hospice Model, which enables family caregivers to have more hands-on roles while being supported by hospice staff and volunteers (Walshe et al., 2021). Within these community-based models, volunteers contribute basic non-clinical support, such as offering companionship and occasional respite for caregivers (Pesut et al., 2014; Overgaard, 2015), while community organizations offer bereavement support, culturally aligned services, transportation, and home-based assistance (Accreditation Canada, 2023; Northern Ontario School of Medicine, 2019; Payne et al., 2017; World Health Organization, 2016). These approaches alleviate non-clinical workload, including psychosocial support, administrative coordination, and household-related tasks, while preserving clinical responsibilities for regulated providers. Reported outcomes included reduced caregiver burden, strengthened community integration, and more sustainable staffing models in resource-constrained settings (He et al., 2020; Payne et al., 2017; Ryan et al., 2019).
  2.3) Meaning-centred spiritual care is another promising approach, as it prioritizes support for both patients experiencing existential distress and staff facing compassion fatigue (Healthcare Excellence Canada, 2023; Kang et al., 2021; Sainz et al., 2019; World Health Organization, 2016). Studies described spiritual care training programs delivered to interdisciplinary teams to enhance their ability to provide emotional, existential, and spiritual support (Kang et al., 2021; Sainz et al., 2019). These programs emphasized reflective practice, structured conversations around meaning, values, and legacy, and skill-building for navigating emotionally complex discussions. Kang et al. (2021) demonstrated increased spiritual care competencies and reduced compassion fatigue among staff immediately following training, with partial retention of skills at follow-up. Implementation was most applicable in settings where psychological or spiritual distress was common, and outcomes included improved patient communication, enhanced holistic care, and reduced emotional strain on staff (Kang et al., 2021).
  2.4) Integrating technology, such as telemedicine and electronic health records, was also identified as a valuable strategy to optimize staffing efficiency, streamlining communication, reducing administrative burdens, and improving coordination among interdisciplinary teams (Blinderman et al., 2021; Central Health Line, 2025; Gurian et al., 2019; Shugarman et al., 2023). Telemedicine enabled remote symptom reviews, interdisciplinary case discussions, and caregiver consultations, reducing staff travel time and facilitating timely clinical decision-making, particularly in rural and home-based programs (Blinderman et al., 2021; Tupala et al., 2020). Electronic health records improved workflow by streamlining documentation, enabling real-time information sharing, and reducing duplication of tasks among interdisciplinary teams (Accreditation Canada, 2019; Wan et al., 2023). Studies reported that these tools helped redistribute staff time toward direct care, improved coordination across dispersed teams, and enhanced responsiveness to urgent needs, thus strengthening service delivery and contributing to workforce sustainability (Accreditation Canada, 2019; Shugarman et al., 2023; Tupala et al., 2020; Wan et al., 2023).
3) *Volunteer Integration*
  The integration of volunteers into multidisciplinary hospice care teams is a crucial theme that emerges from the literature. Several of the reviewed articles outline the diverse contributions of volunteers, emphasizing their roles in providing emotional support, personalized care, patient and family advocacy, practical assistance, community outreach, and the promotion of hospice principles (Claxton-Oldfield and Jones, 2013; Overgaard, 2015; Pesut et al., 2014; Walshe et al., 2021). These contributions are especially valuable in resource-limited settings, as demonstrated by studies showing that volunteer engagement can help address staffing shortages and improve overall patient care experiences and outcomes (He et al., 2020; Terjung et al., 2021;Walshe et al., 2023). The literature also highlights that strong volunteer programs depend on intentional recruitment and training processes, such as Objective Structured Clinical Examination-based assessments shown to predict volunteer readiness and long-term engagement, and retention strategies grounded in intrinsic motivation, clear expectations, and alignment with hospice philosophy (Claxton-Oldfield and Jones, 2013; Szeliga and Mirecka, 2018). Interprofessional collaboration supports this work through models such as Social Model Hospice and Cottage Hospice, where volunteers participate in care within defined scopes and under structured supervision that promotes safety and continuity alongside clinical team members (Grant and Scott, 2015; Walshe et al., 2021).

Despite these benefits, several challenges to sustaining volunteer services are reported. These challenges include inconsistent training, organizational uncertainty about volunteer roles (Pesut et al., 2014), regulatory constraints that limit volunteer involvement in some jurisdictions (Overgaard, 2015), and COVID-19 pandemic-related reductions in volunteer deployment that increased staff burden and reduced patient support (Walshe et al., 2023).

## 4 Discussion

This scoping review synthesized a broad collection of evidence from 47 articles across 11 countries to describe how hospice workforce structures are organized and where improvements may enhance care delivery. While the literature was diverse in geography and methodology, several consistent themes emerged that illustrate how workforce composition, interdisciplinary collaboration, and organizational strategies shape the quality and continuity of hospice care.

The challenges identified in the introduction, including high turnover, inadequate preparatory training, heavy workload, and rural workforce barriers, were consistently reflected across the included studies. The strategies synthesized in this review represent practical responses to these ongoing issues. A clear trend toward flexible, team-based care was evident, with many studies emphasizing the value of interdisciplinary teams that include not only nurses and physicians but also social workers, chaplains, therapists, and volunteers. The most effective strategies were those tailored to patient complexity, emphasizing the need to move beyond fixed staff-to-patient ratios. Ongoing education and training in areas such as spiritual care, cultural competence, and communication were also linked to improved staff retention and improved care experiences for patients and families.

Beyond interdisciplinary collaboration, several articles highlighted the benefits of key approaches to hospice care. Community-led, patient-centred, and spiritually grounded frameworks demonstrated how aligning staffing approaches with the holistic nature of hospice work can improve outcomes and satisfaction. In this context, technology emerged not as a substitute for staff, but as a strategic support, particularly in reducing administrative burden and expanding access in remote settings. Likewise, when integrated thoughtfully and trained adequately, volunteers played a pivotal role in enhancing care delivery and extending the capacity of professional teams.

The feasibility of implementing these strategies varied substantially across settings. Flexible staffing and interdisciplinary approaches were more achievable in inpatient or urban hospices with stable staffing pools, whereas rural programs faced persistent shortages, geographic dispersion, and limited access to specialized providers. Community-based and volunteer-enhanced models were easier to implement in regions with strong social infrastructure, while technology-enhanced care required reliable staff training and organizational investment. These contextual considerations highlight the need for adaptable rather than uniform staffing solutions.

### 4.1 Limitations of the literature

Although this body of literature provides valuable insights, several limitations must be acknowledged. The most prominent is the overrepresentation of articles from the United States, which may bias the applicability of findings to international contexts. In the United States, hospice care is shaped by Medicare regulations and a medicalized model of eligibility, requiring a life expectancy of six months or less, as well as the cessation of curative treatments (Bekelman et al., 2016; Ernecoff and Anhang Price, 2023; Harrison and Connor, 2016). This framework introduces regulatory and insurance-driven barriers to access and often aligns hospice care with the institutional healthcare system. While the United States has expanded access to in-home hospice, reflected in lower rates of in-hospital death, those who enter institutional care are often more likely to receive intensive, high-cost care at the end of life (Bekelman et al., 2016; Khaki et al., 2020; Remington and Wakim, 2010). These patterns are influenced by healthcare financing structures, cultural attitudes toward aggressive care, and systemic incentives (Bekelman et al., 2016; Khaki et al., 2020; Ochman, 2021). In contrast, many other countries frame hospice as a social service, offering access earlier in the illness trajectory and emphasizing community-based care integrated within broader social supports (Bekelman et al., 2016; Ernecoff and Anhang Price, 2023; Ochman, 2021). These fundamental differences highlight that staffing needs and strategies are deeply context dependent. Therefore, recommendations from this review should not be universally applied but rather tailored to reflect local healthcare systems, financing models, and cultural norms.

Additionally, almost half of the 33 peer-reviewed studies were cross-sectional surveys or qualitative in nature. While these methods offer rich, descriptive insights, they cannot establish causality or long-term impact (Tenny et al., 2025; Wang and Cheng, 2020). There is also limited discussion of the economic implications of different staffing strategies. Without data on cost-effectiveness, organizations may find it difficult to determine which strategies are financially sustainable. Furthermore, equity considerations were largely absent. Few articles explored how staffing models affect access to hospice care for marginalized or underserved populations. Considering greater attention to health disparities and the social determinants of health, this represents a critical gap that future research should prioritize.

### 4.2 Limitations of the review

The design of this review, while comprehensive in scope, introduces several limitations. Only four databases (Ovid MEDLINE, EBSCOhost CINAHL, Ovid Embase, and Google Scholar) were searched because it was determined through consultation with the Health Sciences Research Librarian (AR-W) that they are standard repositories used in health sciences research and provide broad coverage of palliative care and nursing. However, we acknowledge that not searching additional databases (e.g., Scopus, Web of Science, ProQuest) may have resulted in relevant studies being missed.

Additionally, by including a wide range of study types and sources, including grey literature, the review aimed to capture a broad view of hospice staffing. However, this inclusivity also led to considerable variability in study quality, populations, and outcomes, making it difficult to draw precise or comparative conclusions. While thematic analysis helped with high-level organization of the findings, the diversity of the data limited the ability to quantify trends or assess effectiveness across settings.

Our search strategy was also restricted to literature published in the English language, which may have excluded other linguistic and cultural contexts; particularly those that involve difference hospice care traditions or emerging staffing models. As a result, the findings may have limited generalizability to the global landscape of hospice staffing. There was also no formal appraisal of study quality conducted as part of this scoping review, which may have influenced the reliability and robustness of the synthesized evidence (Shaheen et al., 2023). While the review strategy allowed for the inclusion of a broad evidence base, it also means that some findings may come from sources with methodological limitations that were not evaluated in detail.

### 4.3 Implications for practice

The review offers practical insights that can inform staffing practices in hospice settings. One of the clearest takeaways is the importance of flexibility in staffing, both in terms of numbers and skill mix. Staffing models that can adapt to patient acuity and complexity are more likely to support continuity of care and reduce staff burnout. Organizations should consider moving away from one-size-fits-all ratios in favour of models that account for workload, scope of practice, and the specific needs of the populations being served in their communities.

In rural or remote contexts, telehealth, cross-trained interdisciplinary teams, and structured volunteer roles may help mitigate persistent staffing shortages. In inpatient settings, maintaining appropriate staff-to-patient ratios and incorporating psychosocial and spiritual care providers can strengthen symptom management and reduce emotional burden on staff (Artico et al., 2022; Kang et al., 2021). Home-based programs may benefit from streamlined documentation systems, mobile technologies, and partnerships with community organizations to support continuity and responsiveness of care (Wan et al., 2023). Tailoring staffing approaches to local resources and patient needs is essential for improving sustainability and quality across hospice settings.

## 5 Conclusion

As the demand for hospice services continues to rise, the question of how to optimally staff these programs is becoming increasingly urgent. This review provides a wide-ranging look at how organizations around the world are navigating this challenge. While there is no single formula for optimal staffing, the evidence points to a set of guiding principles: the need for adaptable models that reflect patient complexity, the value of interdisciplinary collaboration, the importance of ongoing training and support, and the role of volunteers and technology in extending the reach of care.

Moving forward, researchers and policymakers must consider how these insights can be translated into concrete staffing standards and guidelines. There is also a clear need for more rigorous evaluation of what works in practice, not just in terms of patient outcomes, but in terms of sustainability, cost, and equity. In the meantime, hospice programs can draw on the themes identified in this review to inform staffing decisions that are not only efficient but also grounded in compassion and respect for the unique needs of those at the end of life.

## Funding

Funding support was received through internal funding for Catherine Goldie’s Scientist in Nursing Research Chair provided by Providence Care Centre and Queen’s University in Kingston, Ontario.

## CRediT authorship contribution statement

**Victoria Kao:** Writing – review & editing, Writing – original draft, Visualization, Project administration, Investigation, Formal analysis, Data curation. **Vidhi Patel:** Writing – original draft, Investigation, Data curation. **Kiana Aghakhani Barfeh:** Investigation, Data curation. **Philip Pranajaya:** Writing – review & editing, Investigation, Data curation. **Jayne Kang:** Writing – review & editing, Investigation, Data curation. **Madeleine Wong:** Writing – review & editing, Investigation, Data curation. **Hannah McDonald:** Investigation, Data curation. **Amanda Ross-White:** Writing – review & editing, Methodology, Data curation. **Craig E. Goldie:** Writing – review & editing, Supervision, Conceptualization. **Danielle Kain:** Supervision, Conceptualization. **Sarah Moore-Vasram:** Writing – review & editing, Supervision, Conceptualization. **Catherine L. Goldie:** Writing – review & editing, Supervision, Project administration, Investigation, Data curation, Conceptualization.

## Declaration of competing interest

The authors declare that they have no known competing financial interests or personal relationships that could have appeared to influence the work reported in this paper.

## Figures and Tables

**Table:** 

Publication Details	Article Type	Population Characteristics	Aim / Methods	Key Findings	Key Recommendations	Theme(s) Reflected
Luo et al. (2013); United States	Survey	Nursing staff from 1036 home-based and outpatient hospice care agencies	Used data from the 2007 National Home and Hospice Care Survey to estimate 3-month turnover rates and explore factors associated with turnover among various types of nursing staff.	Hospice agencies experienced varying turnover rates, with larger and for-profit agencies facing higher turnover, while higher staffing levels and certain benefits correlated with lower turnover.	To enhance nursing staff retention, target modifiable factors such as nurse staffing levels, health insurance, and work-support programs.	Staffing Composition and Competency
Claxton-Oldfield & Jones (2013); Canada	Survey	119 direct patient/family care volunteers from 3 home-based and outpatient hospice programs: mean age of 61.7 years, 79.8 % female, and an average service length of 6.3 years	Used the 33-item Volunteer Retention Questionnaire to assess the importance of various factors in influencing the decision to continue volunteering.	Volunteer retention was primarily driven by intrinsic factors such as job satisfaction, adequate training, alignment with the hospice philosophy, clear expectations, and the ability to set boundaries.	To enhance volunteer retention, provide comprehensive support and training, align their work with hospice philosophies, and prioritize intrinsic rewards to foster a sense of purpose and fulfillment.	Volunteer Integration
Stone et al. (2013); United States	Survey	160,720 home health workers, including home health aides, certified nursing assistants, hospice aides, and personal care attendants	Used data from the 2007 National Home Health Aide Survey to analyze differences in worker categories in terms of demographics, compensation, benefits, satisfaction, and retention.	Hospice aides had higher wages, better benefits, and greater job retention compared to other home health workers. However, high turnover remained a challenge for HHAs and personal care attendants, with low wages and lack of benefits being major workforce issues.	To enhance the home health workforce, policymakers should improve wages, benefits, and job stability while addressing training requirements, compensation, and workplace conditions to strengthen retention.	Staffing Composition and Competency
Roberts & Hurst (2013); England	Observational	Inpatients in 23 palliative and hospice care wards	Used data from the adapted National Health Service workforce planning method to evaluate hospice staffing needs, comparing them to National Health Service palliative care units by analyzing bed occupancy, patient dependency, staff activity, service quality, and costs.	Hospice wards were better staffed and provided higher-quality care despite facing heavier workloads. However, significant staffing variations across hospices impacted service efficiency and costs.	To enhance hospice workforce planning, staffing models should be based on patient dependency and workload, with standardized benchmarks to optimize costs and maintain high-quality care.	Staffing Composition and Competency
Pesut et al. (2014); Canada, United States, United Kingdom, Netherlands, and Sweden	Review	Hospice volunteers	Used data from 54 studies published between 2002 and 2012 to analyze hospice volunteer roles, recruitment, training, retention, and outcomes, incorporating qualitative, quantitative, and mixed-method studies.	Volunteers provided emotional, psychosocial, and practical support, enhancing patient well-being, improving family satisfaction, and potentially influencing patient longevity. However, gaps existed in recruitment, training, and retention strategies.	To enhance patient-centered care, more structured volunteer training and support programs are needed, along with better integration into palliative care teams. Future research should rigorously examine volunteers' contributions to patient and family outcomes.	Volunteer Integration
Newton & McVicar (2014); United Kingdom	Mixed Methods	67 palliative care nurses, 6 patients, and 13 carers	Used data from surveys, interviews, and focus groups to evaluate the relevance of the Davies & Oberle (1990) supportive care model in contemporary palliative care settings.	The model remained relevant but required revisions to address workload pressures in hospitals and incorporate two new dimensions (displaying expertise and influencing other professionals) to reflect modern nursing roles.	To enhance care delivery, the model should be updated to include advanced communication skills, decision-making, and advocacy roles, while addressing time constraints to maintain strong patient-nurse connections.	Staffing Composition and Competency; Key Care Approaches
Overgaard (2015); Denmark and Australia	Comparative	41 participants including volunteers, staff, and management from two hospices	Used data from non-participant observation and unstructured interviews to explore the boundaries between professional care workers and volunteers in hospices across different welfare state models.	In Denmark, volunteers were excluded from direct care due to professional occupational closure, while in Australia, they participated within limits set by risk management policies. Two models were identified: the occupational closure model in Denmark and the risk model in Australia.	To enhance care, volunteer roles should be clearly defined within organizational and national welfare contexts, balancing professional boundaries with their added value. Future research should explore volunteer roles in broader healthcare settings.	Volunteer Integration
Grant & Scott (2015); United States	Narrative Review	Hospice patients and their families	Used data to introduce Social Model Hospice as a community-based alternative to traditional medical hospice, examining the gap between desired and actual place of death and how Social Model Hospices can bridge this disparity.	Traditional hospice models relied heavily on family caregivers, which became increasingly unrealistic. The Social Model Hospice provided a home-like environment with 24-hour care, utilizing volunteers and paid staff. Patients and families experienced reduced burden and improved well-being.	To enhance end-of-life care, expanding the Social Model Hospice model could alleviate caregiver burden and improve patient experiences. Further research is needed on its impact on grief, cost-effectiveness, and accessibility for low-income populations.	Key Care Approaches; Volunteer Integration
Braveman et al. (2015); United States	Grey Literature	Palliative care patients from inpatient, home-based, and outpatient facilities.	To provide guidance on developing and implementing palliative care programs.	N/A	To enhance delivery, implement an interdisciplinary model that integrates diverse professionals and strengthens patient-centered care through strategic partnerships and planning.	Staffing Composition and Competency; Key Care Approaches
Blacker et al. (2016); United States and Canada	Review	Hospice and palliative care social workers and educators	Used data to examine the role of hospice and palliative care social workers in interprofessional education and interprofessional practice, highlighting leadership opportunities and barriers to collaboration.	Social workers played a critical role in interprofessional collaboration but faced challenges such as role misunderstandings, power imbalances, and lack of standardized training. Effective teamwork improved patient outcomes and reduced healthcare costs.	To enhance interdisciplinary care, social work education should integrate more interprofessional education opportunities to strengthen teamwork skills, while social workers should actively define their role to improve collaboration and leadership. Future research should measure the impact of social work in interdisciplinary settings.	Staffing Composition and Competency; Key Care Approaches
Washington et al. (2016); United States	Qualitative	100 hospice interdisciplinary team meetings including 73 family caregivers and 78 hospice staff members	Used data from video recordings, interviews, and field notes to examine shared decision-making in hospice team meetings with family caregivers participating via video conferencing, applying content and thematic analysis.	Shared decision-making occurred infrequently due to barriers such as time constraints, unclear role expectations, unaddressed emotional needs, communication skill deficits, and staff absences. Facilitators included hospice philosophy, healthcare trends, interdisciplinary team structure, and designated team leadership.	To enhance caregiver involvement, hospice providers should actively engage family caregivers in shared decision-making rather than merely inviting them to meetings. Training in communication, clear role expectations, and emotional support can improve collaboration, while structured video conferencing may further facilitate caregiver participation.	Key Care Approaches
World Health Organization (2016); Global	Grey Literature	Governments, health program managers, and palliative care providers	To provide guidance on planning and implementing palliative care services at the national, provincial, and district levels. Covers integration into health systems, workforce development, and medication access.	N/A	To enhance care, governments should prioritize policy development, expand access to essential medications, strengthen workforce capacity, and promote interdisciplinary collaboration. Sustainable funding models and integration into primary healthcare are essential for expansion.	Staffing Composition and Competency; Key Care Approaches
Palliative and Hospice Nursing Panel (2017); United States	Grey Literature	Nurses in palliative and hospice care settings	To provide outlined recommendations for nurses to lead and transform palliative care through practice, education, administration, policy, and research. Developed by the American Nurses Association and Hospice and Palliative Nurses Association.	N/A	To enhance nursing, standardize education, integrate palliative care into licensing exams, increase program funding, reduce workforce barriers, and position nurses in leadership and policymaking roles.	Staffing Composition and Competency
Washington et al. (2017); United States	Qualitative	24 hospice providers from 11 agencies, including nurses, chaplains, social workers, physicians, and other hospice staff	Used data from interviews and template analysis, applying the American Congress of Rehabilitation Medicine model of interprofessional practice, to examine team functioning in hospice interprofessional meetings, identifying key barriers and facilitators.	Team meetings supported high-quality, holistic patient care but were often inefficient due to time constraints, large caseloads, and hierarchical team structures. Many team members felt undervalued, and discussions frequently prioritized biomedical issues over psychosocial and spiritual concerns.	To enhance efficiency in hospice care, standardizing meeting processes can streamline discussions by focusing on new and relevant patient information. Inclusive interprofessional communication and smaller team groups can further improve collaboration, engagement, and care outcomes.	Key Care Approaches
Bhavsar et al. (2017); United States	Observational	Hospice and palliative care staff, including physicians, nurse practitioners, registered nurses, social workers, administrative staff, and volunteers	Used data from observations and task recordings across three settings within a hospice-led palliative care network to investigate time spent on clinical and administrative activities. Created a process map to analyze workflow efficiency.	Physicians spent 94 % of their time on patient care, while nurses devoted a significant portion to administrative tasks. Social workers focused entirely on patient care. Although interdisciplinary care involved numerous tasks, inefficient administrative burdens limited direct patient interaction.	To enhance patient interactions, reducing clinicians' administrative workload and standardizing task distribution can improve efficiency. Future policies should provide financial incentives to support community-based palliative care models.	Staffing Composition and Competency; Key Care Approaches
Payne et al. (2017); United Kingdom	Qualitative	34 patients (17 cancer, 10 chronic obstructive pulmonary disease, 7 heart failure), 13 family carers, and 23 healthcare professionals across four hospices	Used data from qualitative interviews to investigate hospice integration with local healthcare providers, analyzing how hospice services support, supplement, or supplant local care.	Hospices complemented local services and, in some cases, replaced them by providing inpatient care. While patients valued continuity, named professionals, and integrated systems, integration was hindered by fragmented communication, lack of coordination, and funding constraints.	To enhance care, efforts should focus on improving information sharing, defining coordination roles, and ensuring continuity of care. Policymakers should address funding stability and workforce shortages, while future research refines models of integrated palliative care.	Key Care Approaches
Szeliga & Mirecka (2018); Poland	Observational	154 hospice volunteer candidates at St. Lazarus Hospice, Krakow	Used data from multiple-choice questions , open-ended questions, and Objective Structured Clinical Examination ,followed by long-term observation, to evaluate methods for selecting and verifying hospice volunteers based on knowledge, skills, and attitudes.	Objective Structured Clinical Examination was the strongest predictor of volunteer success, outperforming multiple-choice questions and written exams. Volunteers with higher Objective Structured Clinical Examination scores were more likely to remain active and perform well in hospice care.	To enhance hospice volunteer selection, structured assessments like Objective Structured Clinical Examinations should ensure competence and suitability, with alternative roles for candidates who struggle in practical exams. Future research should refine Objective Structured Clinical Examination methods for volunteer training.	Volunteer Integration
Dudley et al. (2018); United States	Qualitative	20 leaders from community-based palliative care programs, including academic medical centers, home health, hospice organizations, and the Veterans Administration.	Used data from semi-structured interviews with program leaders to explore staffing, recruitment, and training challenges in community-based palliative care.	Staffing models differed by setting, with academic programs led by physicians and community programs relying on nurse practitioners and registered nurses. A shortage of trained palliative care professionals persisted, with on-the-job training common and gaps in key competencies like communication and independent decision making.	To enhance community-based palliative care programs, standardized training for new hires should include advanced communication skills and interdisciplinary collaboration. Structured training and recruitment strategies are essential to addressing workforce shortages and high turnover rates.	Staffing Composition and Competency
Pawlow et al. (2018); United States	Survey	556 advanced practice registered nurses in hospice and palliative care	Used data from an online survey to examine the workforce composition, educational needs, and barriers to practice for advanced practice registered nurses in hospice and palliative care.	Most advanced practice registered nurses had extensive nursing experience but were relatively new to palliative care roles. Many reported insufficient palliative care training in graduate programs, relying on continuing education. Challenges included a limited scope of practice, lack of role recognition, and regulatory barriers, particularly for clinical nurse specialists (CNSs).	To enhance workforce readiness, standardizing palliative care education in advanced practice registered nurse programs is essential. Regulatory changes should expand advanced practice registered nurse scope of practice in prescribing and billing, while greater role recognition can improve workforce integration and effectiveness.	Staffing Composition and Competency
Head et al. (2019); United States	Survey	633 hospice and palliative nurses, including registered nurses, advanced practice registered nurses, and licensed practical nurses	Used data from an online survey utilizing the Home Healthcare Nurse Job Satisfaction Scale to investigate job satisfaction, stressors, intent to leave, and self-care strategies among hospice and palliative nurses.	Most nurses reported high job satisfaction, yet nearly half frequently considered quitting. Higher salaries correlated with greater job satisfaction, while top stressors included high workload, poor administration, and lack of recognition. Common self-care activities included physical exercise, hobbies, and socializing, though some relied on unhealthy coping mechanisms.	To enhance nurse retention and job satisfaction, employers should improve workplace culture, reduce workload, and increase recognition and salaries. Strengthening support through team wellness programs and better leadership can further improve workforce stability.	Staffing Composition and Competency
Greenstein et al. (2019); United States	Review	Primary care physicians and hospice providers	Used data to examine the role of primary care physicians in hospice care, focusing on the Medicare Hospice Benefit, regulatory guidelines, reimbursement policies, and employment opportunities in hospice settings.	Primary care physicians played a critical role in timely hospice referrals, though uncertainty about prognosis and discomfort with end-of-life discussions often led to delays. Most hospice admissions were for non-cancer diagnoses, and while Medicare regulations required primary care physicians to certify eligibility for reimbursement, many were unaware of the related billing process.	To enhance hospice care, primary care physicians should receive more training on eligibility criteria and end-of-life discussions, while greater collaboration with hospice teams can improve patient transitions. Policymakers should simplify reimbursement structures to encourage primary care physician participation.	Key Care Approaches
Sainz et al. (2019); Canada	Grey Literature	Residential hospice facilities, healthcare policymakers, and providers	To provide residential hospice standards for New Brunswick, developed best practices for governance, clinical care, operations, and quality assurance based on national and provincial frameworks.	N/A	To enhance residential hospice care, alignment with national best practices is essential to ensure patient-centered, culturally competent care. Standardizing referral processes, funding mechanisms, and staffing models is crucial for sustainability, while collaboration with healthcare systems supports integrated palliative care delivery.	Staffing Composition and Competency; Key Care Approaches
Northern Ontario School of Medicine (2019); Canada	Grey Literature	Hospice palliative care providers, patients, families, and healthcare administrators	To provide insights into rural hospice palliative care within acute care hospitals, evaluated effectiveness by surveying providers and administrators on challenges, strengths, and sustainability.	Strengths included adequate staffing ratios, 24/7 pharmacy support, trained palliative care nurses, and strong community integration. Challenges involved stigma around palliative care, funding inequities, limited community-based resources, and workforce shortages.	To enhance rural hospice care, increase funding equity for hospice beds, expand education for staff, families, and caregivers, implement early identification strategies for palliative care patients, and improve community integration and provider coordination.	Staffing Composition and Competency; Key Care Approaches
Gurian et al. (2019); United States	Grey Literature	Medicare beneficiaries eligible for hospice care	To provide insights into hospice care trends, examined differences between nonprofit and for-profit providers, changes in the Medicare Hospice Benefit, and regulatory impacts using policy analysis, industry reports, and financial data.	Nonprofit hospices emphasized community integration, interdisciplinary care, and bereavement services, while for-profit hospices reported higher profit margins and growth rates. Concerns about quality disparities and regulatory oversight included fraud, live discharge rates, and equitable access to all levels of hospice care.	To enhance hospice care, policymakers should support nonprofit hospices with equitable reimbursement, stronger regulations, and greater transparency while increasing oversight of for-profit models to prevent exploitation and ensure patient-centered care.	Staffing Composition and Competency; Key Care Approaches
Accreditation Canada (2019); Canada	Grey Literature	Hospice, palliative, and end-of-life care providers, healthcare administrators, and policymakers	To provide quality standards for hospice and palliative care across Canada, focuses on service excellence, patient safety, workforce competency, and interdisciplinary collaboration.	N/A	To enhance organizations should prioritize interdisciplinary teamwork, standardized volunteer training, and sustainable funding models. Enhancing data systems, patient engagement, and evidence-based practice can improve care outcomes. Policymakers should address workforce gaps and ensure equitable access to palliative care.	Staffing Composition and Competency; Key Care Approaches
Ryan et al. (2019); Ireland	Grey Literature	People with life-limiting or life-threatening illnesses	Collaborated with healthcare providers, patients, carers, and advocacy organizations to design a model of palliative care that prioritizes equitable access, regardless of care setting or diagnosis.	N/A	To integrate palliative care into healthcare systems, organizations should prioritize interdisciplinary teamwork, standardized training, sustainable funding, and evidence-based practices, while policymakers should address workforce gaps and equitable access.	Key Care Approaches
He et al. (2020); United States	Review	Hospice agencies and patients receiving hospice care	Conducted a systematic review of empirical research on hospice performance using structure-process-outcome and structure-conduct-performance frameworks to understand how hospice performance is measured, and what factors are considered.	Hospice agencies adopted strategies related to service provision and staffing to enhance performance, with practices such as volunteer use being linked to better outcomes. While most studies examined the relationship between hospice structure and strategy, fewer explored structure-performance, and even fewer investigated strategy-performance.	To develop standardized hospice performance measures based on patient experiences, future research should examine market, patient, and organizational factors, explore the structure-performance relationship, and identify innovative practices to enhance quality.	Key Care Approaches; Volunteer Integration
Tupala et al. (2020); Canada	Grey Literature	52 specialist palliative care nurses working in community-based palliative care programs	Used an anonymous online questionnaire that covered time allocation for patient care and non-patient care tasks, factors influencing care time, and expected future changes to estimate the number of palliative care program nurses needed for the next 20 years in Nova Scotia.	Nurses spent 60 % of their time on direct patient care, 20 % on educational and administrative tasks, and 20 % on travel. Factors influencing time spent on patient care included disease severity, patient education needs, and the availability of family support. Nurses anticipated increased workload and complexity in future palliative care roles.	To maximize workforce capacity and patient care time, especially in rural areas, hospices should use operations research for planning, consider diverse patient needs, and strengthen the integration of education and administrative support.	Staffing Composition and Competency
Blinderman et al. (2021); United States	Retrospective Review	Patients requiring palliative care at New York-Presbyterian hospitals during March and April 2020	Used administrative data, and family and physician feedback to report on innovative care models deployed during the COVID-19 pandemic to meet rising palliative care demands, highlighting successes, challenges, and future directions.	The models successfully expanded access to palliative care, with virtual outpatient visits, embedded staff, interdisciplinary team expansion, hospice units, and ePalliative Care services being particularly effective in meeting the increased demand.	To strengthen palliative care during and beyond the COVID-19 pandemic, flexibility and adaptability should be prioritized in interventions to improve patient access, workforce expansion, and staff comprehension.	Key Care Approaches
Hasson et al. (2021); United Kingdom	Qualitative	35 hospice management and staff involved in the delivery of palliative care day services at 3 hospices	Used thematically analyzed data from 6 focus groups to explore the strengths and challenges of palliative day care services from the perspectives of nurses, consultants, allied health professionals, healthcare assistants, and volunteers.	Four main themes emerged, revealing that palliative day services are shifting from a social to a more medical model: variations in care models; unclear referrals and inconsistent patient populations; recognition of strengths and challenges; and an uncertain future for day services.	To improve access, consistency, and impact, future efforts should standardize referral processes, integrate medical and social care, advocate for funding and visibility, and promote early referrals for palliative day services.	Key Care Approaches
Walshe et al. (2021); England	Qualitative	26 hospice staff, volunteers, family caregivers, patients, and external stakeholders involved in the development of the Cottage Hospice model	Used a participatory action research design, including situational analysis and action cycles, with interviews and document analysis (2017–2018) to explore the development of this new model for end-of-life care, specifically focusing on the role of family caregivers and volunteers.	Three main themes emerged: imagining the future of Cottage Hospice to meet the growing demand for hospice care in a home-like setting; developing the role of family caregivers; and quality and safety issues particularly regarding the reliance on volunteers.	To enhance home-based end-of-life care, future efforts should actively involve and support family caregivers and volunteers, establish formal caregiver agreements, assess the feasibility of volunteer-led models, and explore replicating the Cottage Hospice approach.	Key Care Approaches; Volunteer Integration
McInnerney et al. (2021); United Kingdom	Survey	Hospice staff, including clinical psychologists, counselors, and other members of the palliative care team, from 116 hospices	Used an online survey with quantitative and qualitative questions to assess the availability, organization, and adequacy of psychological services, as well as identify facilitators and barriers to effective service delivery.	The survey found improved access to psychological services compared to previous surveys, but only 16 % of hospices deemed specialized care for complex needs as adequate, citing funding, staffing shortages, and inconsistent referrals as key barriers.	To improve access to specialized psychological support in hospice care, efforts should focus on developing external partnerships, clearer referral pathways, staff training, service audits, and timely care for complex needs.	Staffing Composition and Competency; Key Care Approaches
Kang et al. (2021); South Korea	Quasi-Experimental	13 hospice palliative care team members, including nurses, social workers, and professionals from related fields	Used a pretest-posttest design to evaluate a meaning-centred spiritual care training program that was developed to enhance hospice palliative care teams’ abilities to provide spiritual care to patients.	The study found significant improvements in compassion fatigue, spiritual care competencies, and spiritual care therapeutics immediately after the intervention. At the four-week follow-up, only spiritual care competencies showed sustained improvement.	To enhance the spiritual component of hospice palliative care, efforts should integrate programs like meaning-centred spiritual care training in routine staff training, emphasize self-reflection and compassion, ensure ongoing evaluation of spiritual care techniques, and adapt to cultural differences.	Staffing Composition and Competency; Key Care Approaches
Artico et al. (2022); Italy	Observational	992 adult patients with life-threatening illnesses (both oncological and non-oncological) in 13 residential hospices	Used a secondary analysis of data collected from a multicenter, prospective longitudinal observational study conducted between November 2017 and September 2018 to explore the relationship between staffing models and the control of clinically significant symptoms.	The optimal staffing model for controlling symptoms included a patient-to-physician ratio of 5.5:1–6.5:1, a patient-to-nurse ratio of 1.5:1–2.7:1, and a patient-to-nurse-assistant ratio of 4.1:1–6.3:1. Hospices with 12–25 beds and a median stay of 12 days showed a significantly higher likelihood of controlling symptoms effectively.	To optimize palliative care, efforts should adopt balanced staffing models, ensure adequate hospice bed availability and length of stay, provide specialized training, and conduct further research to refine best practices.	Staffing Composition and Competency
Chapman et al. (2022); England	Qualitative	61 healthcare professionals, including doctors, nurses, and allied healthcare professionals working in inpatient, outpatient, and community settings in 5 hospices	Used qualitative semi-structured focus groups and interviews, conducted between May and November 2019, to explore barriers and facilitators to effective symptom management in palliative care settings.	A novel model of symptom management emerged, featuring a coordinated, multi-step approach with engagement, partnership, decision-making, and delivery. Psychological distress was identified as a barrier to effective symptom management, and family involvement was crucial for ensuring the effective interventions.	To improve symptom management in palliative care settings, efforts should focus on early identification of psychological distress, shared decision-making with patients and families, enhanced communication with multidisciplinary teams, and the use of non-medical interventions like informal psychological support.	Key Care Approaches
Walshe et al. (2023); Multi-national (35 countries)	Survey	Those responsible for managing volunteers at 304 palliative care organizations	Used an online survey distributed from May to July 2021, to understand how the roles and deployment of volunteers within specialist palliative care services changed during the COVID-19 pandemic.	The survey found that 47.7 % of organizations had changed their volunteer deployment since the pandemic, with a significant reduction in volunteers, especially those in direct patient-facing roles. The reduction in volunteer numbers led to increased workload on staff and contributed to patient isolation and decreased support.	To adapt to changing palliative care needs, efforts should include creating new roles, recruiting and retaining younger volunteers, integrating virtual opportunities, and providing targeted infection and prevention control training, especially for the COVID-19 pandemic.	Volunteer Integration
Melekis et al. (2023); United States	Retrospective Review	500 terminally ill patients in community-run residential care homes: 63.6 % women, 94.9 % Caucasian, mean age of 77.8, and 71.6 % had cancer diagnoses	Used a retrospective chart review to explore the explore the demographic, pre-admission housing, and caregiving characteristics of patients receiving care in residential care homes. There was a particular focus on patients with housing insecurity and/or caregiver instability.	Most patients came from private homes or hospitals and had prior caregiver support, but nearly half lived alone. The length of stay at the residential care home was short, with 50 % dying within 10 days of admission.	To improve care, efforts should explore expanding Social Model Hospice residential care homes for patients with housing or caregiver instability, refine referral processes for equitable access, enhance hospice agencies’ roles in increasing awareness, and investigate whether residential care homes improve hospice utilization and quality of life for terminally ill patients.	Key Care Approaches; Volunteer Integration
Wan et al. (2023); United States	Cohort	5418 adults aged 66 years and older who were diagnosed with metastatic breast cancer for the first time between 2002 and 2009, and died between 2002 and 2010	Used Surveillance Epidemiology End-Results—Medicare linked data 2000–2010, Medicare Provider of Service files, and Census 2000 U.S. Zip Code Tabulation Areas files to examine the relationship between local hospice capacity, staffing levels, and hospice use, with a focus on rural versus urban disparities.	The study found that rural and suburban areas, especially those distant from metropolitan areas, had lower hospice use. Increased hospice staffing in these areas was associated with higher hospice use, while the number of hospice programs did not have a significant impact.	To improve end-of-life care in rural and underserved areas, efforts should increase hospice staffing, address access barriers through telehealth and interprofessional teams, enhance public awareness, and advance research on rural healthcare disparities and innovative care models.	Staffing Composition and Competency; Key Care Approaches
Cornell et al. (2023); United States	Cohort	43,200 rural veterans who received primary care services from a site enrolled in the Social Work Patient Aligned Care Team staffing program and who were recently hospitalized	Used difference-in-differences analyses of the change in palliative care use with the implementation of the Social Work Patient Aligned Care Team staffing program at 71 Veterans Affairs primary care sites, between October 2016 and September 2019, to assess the association between social work staffing and palliative care use among veterans.	The study found that after the addition of social workers, the use of palliative care among veterans significantly increased, nearly doubling compared to before the intervention.	To expand palliative care access, especially for rural veterans, efforts should increase social work staffing in primary care, explore their role in facilitating discussions and referrals, and evaluate long-term impacts on health outcomes and healthcare use.	Staffing Composition and Competency; Key Care Approaches
Shugarman et al. (2023); United States	Mixed Methods	82 hospice providers and 2294 beneficiaries who voluntarily participated in the Medicare Care Choices Model.	Used a descriptive analysis of qualitative survey data, service data, and Medicare administrative data to examine how hospices responded to the COVID-19 pandemic and the impacts on beneficiaries.	The study found that most hospices shifted to telehealth to reduce in-person visits, leading to increased phone-based encounters, decreased in-person services, and fewer total encounters per beneficiary.	To strengthen future hospice care, efforts should expand telehealth use, train staff in virtual care and infection control, address in-person service declines, and assess the long-term impact of the COVID-19 pandemic on service delivery.	Key Care Approaches
Healthcare Excellence Canada & Canadian Partnership Against Cancer (2023); Canada	Grey Literature	The hospice team at Kensington Hospice, a 19-bed facility located in downtown Toronto	To report on the development and implementation of an equity-oriented model of hospice care that emphasizes low-barrier access for structurally vulnerable populations.	Kensington Hospice successfully integrated equity-focused care, serving 35–50 % structurally vulnerable patients, exceeding its target of 25 %.	To ensure equitable hospice care for vulnerable populations, efforts should adopt low-barrier, trauma-informed, and culturally safe practices, expand harm reduction programs, and strengthen community partnerships.	Key Care Approaches
Accreditation Canada (2023); Canada	Grey Literature	Clients receiving home care, hospice, palliative, and end-of-life services in Alberta	From May 29 to June 2, 2023, data was gathered through on-site surveys and interviews with administration, clients and their families, staff, and other stakeholders to complete the accreditation process.	There was 90 % compliance with assessed criteria, marked by client-centred care, timely access for complex cases, strong teamwork, and wound care expertise. Rural staff recruitment and retention remained a challenge.	To strengthen service delivery, focus on staff retention, workload management, virtual care expansion, standardized processes, knowledge sharing, client and family engagement, change management, and measurable quality improvement.	Staffing Composition and Competency; Key Care Approaches
Terjung et al. (2021); United States, Sweden, Belgium, United Kingdom, and Australia	Review	Adult patient receiving palliative or hospice day-care, staff members, volunteers, family members/caregivers, and referrers	Used scoping review methodology for the analysis 32 studies published until June 2020, to map the status, demand, and practice models of palliative day-care clinics and day hospices.	The review found that while these services are valued for their multidisciplinary approach, they vary in delivery, referrals, and patient populations, with underrepresentation of younger, non-malignant, and ethnic minority patients.	To strengthen palliative day-care and day hospice services, efforts should focus on patients who would benefit most, improving provider education to reduce misconceptions, and addressing funding challenges for sustainability and growth.	Key Care Approaches
Shalev et al. (2024); United States	Survey	708 hospice and palliative medicine clinicians that are members of the American Academy of Hospice and Palliative Medicine	Used a descriptive analysis of online survey data to explore the frequency of encounters with common psychiatric comorbidities and how they are managed by hospice and palliative medicine clinicians.	Hospice and palliative medicine clinicians commonly manage complex psychiatric comorbidities (particularly mood, anxiety, and neurocognitive disorders), despite low comfort levels, limited access to specialists, underuse of evidence-based screening tools, and insufficient training opportunities.	To strengthen mental health care in palliative settings, integrate mental health services into care models, embed specialists within teams, expand clinician training, streamline referrals, and empower social workers to provide mental health support.	Staffing Composition and Competency; Key Care Approaches
National Hospice and Palliative Care Organization (2024); United States	Grey Literature	Hospice providers, including nurses, physicians, social workers, chaplains, personal care aides, and administrative staff	To provide hospices with a framework for assessing staffing levels based on organizational needs, patient populations, and care delivery models.	N/A	To ensure stable, high-quality hospice care, staffing models should be guided by a framework that incorporates patient acuity, psychosocial complexity, and organizational factors, uses full-time hospice-certified staff, and is regularly reviewed to reflect evolving care needs.	Staffing Composition and Competency
Fredericks (2024); Canada	Grey Literature	Nurses, including registered nurses, licensed practice nurses, and nursing assistants	To explore five different nurse staffing models, offering insights into how they impact patient care and nurse workload. It presents the models of team nursing, primary nursing, and total patient care, floating staffing, and patient-acuity based staffing.	N/A	To optimize efficiency and patient outcomes, facilities should select a nurse staffing model aligned with their care needs and structure, ensure staff are well-trained and informed, and regularly evaluate the model’s effectiveness.	Staffing Composition and Competency
Central Health Line (2025); Canada	Grey Literature	Volunteers and healthcare professionals in community and residential hospices	The aim is to provide palliative care services through community-based hospices and residential hospices. Methods include home visits, telephone support, grief and bereavement counseling, and other specialized services tailored to patients' needs.	N/A	To enhance palliative care access and support, hospices should consider providing free residential care for patients with a 1–3-month prognosis, engage in community outreach and education, support caregivers and those experiencing anticipatory grief, and maintain strong partnerships with healthcare providers and volunteer programs.	Key Care Approaches; Volunteer Integration
